# Digital Exclusion and Depressive Symptoms among Older People: Findings from Five Aging Cohort Studies across 24 Countries

**DOI:** 10.34133/hds.0218

**Published:** 2025-01-10

**Authors:** Jingjing Wang, Xinran Lu, Sing Bik Cindy Ngai, Lili Xie, Xiaoyun Liu, Yao Yao, Yinzi Jin

**Affiliations:** ^1^ Department of Global Health, School of Public Health, Peking University, Beijing, China.; ^2^ China Center for Health Development Studies, School of Public Health, Peking University, Beijing, China.; ^3^ Mental Health Education and Guidance Center, Department of Student Affairs, Nanjing University of the Arts, Nanjing, China.; ^4^ Department of Global Health and Population, Harvard T.H. Chan School of Public Health, Boston, MA, USA.; ^5^ Department of Chinese and Bilingual Studies, The Hong Kong Polytechnic University, Hong Kong, China.; ^6^ Center for Population and Development Studies, Renmin University of China, Beijing, China.; ^7^ Key Laboratory of Epidemiology of Major Diseases (Peking University), Ministry of Education, Beijing, China.; ^8^ State Key Laboratory of Vascular Homeostasis and Remodeling, Peking University, Beijing, China.

## Abstract

**Background:** Digital exclusion is a global issue that disproportionately affects older individuals especially in low- and middle-income nations. However, there is a wide gap in current research regarding the impact of digital exclusion on the mental health of older adults in both high-income and low- and middle-income countries. **Methods:** We analyzed data from 5 longitudinal cohorts: the Health and Retirement Study (HRS), the English Longitudinal Study of Aging (ELSA), the Survey of Health, Ageing and Retirement in Europe (SHARE), the China Health and Retirement Longitudinal Study (CHARLS), and the Mexican Health and Aging Study (MHAS). These cohorts consisted of nationwide samples from 24 countries. Digital exclusion was defined as the self-reported lack of access to the internet. Depressive symptoms were assessed using comparable scales across all cohorts. We used generalized estimating equation models, fitting a Poisson model, to investigate the association between the digital exclusion and depressive symptoms. We adjusted for the causal directed acyclic graph (DAG) minimal sufficient adjustment set (MSAS), which includes gender, age, retirement status, education, household wealth, social activities, and weekly contact with their children. **Results:** During the study period (2010–2018), 122,242 participants underwent up to 5 rounds of follow-up. Digital exclusion varied greatly across countries, ranging from 21.1% in Denmark to 96.9% in China. The crude model revealed a significant association between digital exclusion and depressive symptoms. This association remained statistically significant in the MSAS-adjusted model across all cohorts: HRS [incidence rate ratio (IRR), 1.37; 95% confidence interval (CI), 1.28 to 1.47], ELSA (IRR, 1.32; 95% CI, 1.23 to 1.41), SHARE (IRR, 1.30; 95% CI, 1.27 to 1.33), CHARLS (IRR, 1.62; 95% CI, 1.38 to 1.91), and MHAS (IRR, 1.31; 95% CI, 1.26 to 1.37); all *P*s < 0.001. Notably, this association was consistently stronger in individuals living in lower wealth quintile households across all 5 cohorts and among those who do not regularly interact with their children, except for ELSA. **Conclusions:** Digital exclusion is globally widespread among older adults. Older individuals who are digitally excluded are at a higher risk of developing depressive symptoms, particularly those with limited communication with their offspring and individuals living in lower wealth quintile households. Prioritizing the provision of internet access to older populations may help reduce the risks of depression symptoms, especially among vulnerable groups with limited familial support and with lower income.

## Introduction

Depression is a major challenge among aging populations and a primary cause of disability, substantially contributing to the worldwide burden of illness [1]. Approximately 13.3% of older adults worldwide experience major depression, highlighting the need for enhanced focus on depression prevention [2]. It is crucial to recognize modifiable risk factors for depression to initiate effective early interventions.

The digital exclusion refers to the disparities in access to and use of information and communication technologies, including the internet, which has emerged as a major concern [3]. Internet use has been associated with improved health outcomes and social well-being in older populations. This offers potential ways to combat loneliness and depression, particularly as social connections decrease with age [4,5]. The COVID-19 pandemic and associated lockdown measures have worsened social isolation and widened the digital exclusion among older adults [6].

Digital exclusion is increasingly recognized as a modifiable risk factor for depression. A study utilizing data from a nationally representative cross-sectional survey in China provides evidence on the relationships between various dimensions of the digital divide—specifically the access divide, support divide, and usage divide—and the prevalence of depressive symptoms among older adults [7]. Additionally, consistent and new internet users have shown a positive correlation with a reduction in depressive symptoms [8].

Despite the potential benefits of internet access, older adults make up a notable proportion of non-users globally, particularly in developing nations; digital exclusion rates are 21.69% in Denmark, 22.61% in the United Kingdom, 60.78% in Mexico, and 97.15% in China [9]. Furthermore, the China Longitudinal Aging Social Survey indicates that only 11.3% of older adults utilize the internet, thereby exacerbating existing social and health disparities [10,11]. However, prior studies investigating the relationship between the digital exclusion and depressive symptoms have yielded inconclusive results [12–15]. Some studies have found that internet use decreases the likelihood of experiencing a depressive state among older adults using longitudinal or cross-sectional analysis [12,14,15]. In contrast, one study on older American cancer survivors found no correlation between information technology use and depressive symptoms, using a cross-sectional and small sample study [13]. To reconcile these discrepant findings, we utilized data from 5 cross-country longitudinal studies to assess the broader consistency of the results.

Moreover, older adults can benefit from close intergenerational relationships, which help them feel more secure and develop coping skills [16,17]. Bridging digital divide may be beneficial for those who live alone or lack support and contact with their children [18]. In fact, many individuals who do not use the internet independently develop strategies to access online content by engaging members of their social support networks who are proficient internet users [19,20]. Intergenerational care has a substantial positive impact on the mental health of older adults [21], with emotional support and daily care positively influencing their mental well-being [22]. However, few studies have explored the mechanism of intergenerational relationship in the context of internet use and mental health. One study identified a moderating effect of intergenerational relationships on the association between internet usage and mental well-being [17]. Additionally, previous research has shown a statistically significant interaction between internet use and irregular contact with their children. The depressive symptoms observed in older adult individuals living alone are associated with intergenerational support, and internet usage may mitigate the risk of depressive symptoms resulting from insufficient intergenerational support [23].

Income level also plays a crucial role in digital exclusion. Individuals with lower income exhibit reduced rates of internet usage. One study revealed that among low-income, homebound individuals, those with lower incomes were significantly less likely to use the internet, underscoring the enduring adverse impact of low socioeconomic status on digital inclusion [24]. This study employed income level as a moderating variable to examine the relationship between digital exclusion and depressive symptoms among older adults across varying income brackets, given its strong influence on individual subjective well-being [25].

To fill the research gaps, we conducted a longitudinal analysis on 5 extensive comparative cohort studies involving older adults from 24 countries in both high-income countries [high-income countries (HICs): Health and Retirement Study (HRS), English Longitudinal Study of Aging (ELSA), and Survey of Health, Ageing and Retirement in Europe (SHARE)] and low- and middle-income countries [low- and middle-income countries (LMICs): China Health and Retirement Longitudinal Study (CHARLS) and Mexican Health and Aging Study (MHAS)]. With this design, we aim to demonstrate broader consistency in the association between digital exclusion and depressive symptoms. Additionally, subgroup analyses were conducted to identify populations that may be more vulnerable to digital exclusion. The present study proposed 2 hypotheses: (a) Digital exclusion is associated with depressive symptoms among older individuals across all 5 cohorts. (b) The relationship between digital exclusion and depressive symptoms varies depending on the level of contact with one’s children and income.

## Methods

### Study design and participants

This study recruited participants from 5 international longitudinal cohorts on aging: HRS (United States), ELSA (England), SHARE, CHARLS, and MHAS. Further details have been described in previous studies [26–30]. All the studies provide information on measuring digital exclusion and its correlation with depressive symptoms. Our study compares data from 5 international cohorts using a similar time period: HRS (2010–2018), ELSA (2010–2018), SHARE (2011–2019), CHARLS (2011–2018), and MHAS (2012–2018).

We included observations that met the following criteria: (a) self-completed surveys without the use of proxies; (b) participants from countries with at least one follow-up survey; (c) individuals aged 60 and above at the time of the survey; and (d) those with complete data on depressive symptoms, digital exclusion, and relevant covariates. The final sample includes participants from 24 countries. A total of 13,886 participants (24,024 person-waves) from HRS, 9,079 participants (26,866 person-waves) from ELSA, 74,933 participants (158,244 person-waves) from SHARE, 11,832 participants (26,018 person-waves) from CHARLS, and 12,512 participants (27,239 person-waves) from MHAS were accessible for the final analyses (comparison between included and excluded observations: Table S2).

### Procedure

The measurements of exposure (digital exclusion), outcome (depressive symptoms), and covariates (gender, age, retirement status, education, household wealth, social activities, and contact with children) are replicated in all 5 cohorts.

We collected data on digital exclusion through self-reported questionnaires. In HRS, digital exclusion was evaluated with the question: “Do you regularly use the Internet (or the World Wide Web) for sending and receiving e-mail or for any other purpose, such as making purchases, searching for information, or making travel reservations?”. In ELSA, participants were asked their frequency of internet use, with responses ranging from 1 = “Every day, or almost every day” to 6 = “never”. In SHARE, digital exclusion was measured by asking: “In the last 7 days, have you used the Internet at least once for e-mailing, searching for information, making purchases, or for any other purpose?”. In CHARLS, digital exclusion was assessed with the question: “Have you used the Internet in the past month?”. MHAS did not have an individual-level question about the digital exclusion, but instead asked “Do you have Internet access at home?”. The term digital exclusion was used to refer to respondents who answered “no” (HRS, SHARE, CHARLS, and MHAS) or reported using the internet less than once a week (SHARE), while digital inclusion referred to those who answered “yes” or reported using the internet at least once a week.

Depressive symptoms were assessed using self-reported questionnaires. In both HRS and ELSA, participants were asked to respond with “yes” (1) or “no” (0) to 8 items from the Center for Epidemiological Studies—Depression (CES-D) scale. These items assessed symptoms experienced over the past week, including “depressed; everything was an effort; sleep was restless; happy (reverse coded); lonely; sad; enjoyed life (reverse coded); and could not get going”, with a score range of 0 to 8. In SHARE, the 12-item EURO-D scale was used to assess depressive mood. Participants were asked to respond with “yes” (1) or “no” (0) to the following question “depressed mood, pessimism, wishing death, guilt, sleep, interest, irritability, appetite, fatigue, concentration, enjoyment and tearfulness”. The CES-D and EURO-D scales measure similar dimensions and is capable of distinguishing depressive symptoms [31]. In CHARLS, depressive symptoms were assessed using the 10-item Center for Epidemiologic Studies Depression Scale (CES-D-10). Participants were asked to recall experiences from the past weeks, including “being bothered by things that do not usually bother, having trouble concentrating, feeling depressed, feeling that everything you did was an effort, feeling hopeful about the future (reverse coded), feeling fearful, being sleepless, feeling happy (reverse coded), feeling lonely, and could not get going”. Responses ranged from 0 = “rarely”, 1 = “some days” (1 to 2 days), 2 = “occasionally” (3 to 4 days), to 3 = “most of the time” (5 to 7 days), with a score range of 0 to 30. In MHAS, depressive symptoms were assessed using the 9-item CES-D scale. Participants recalled past week experiences, responding with yes(1) or no(0) to item including feeling depressed, feeling everything was an effort, restless sleep, feeling happy (reverse scored), feeling lonely, enjoyed life (reverse scored), feeling sad, feeling tired, and having a lot of energy (reverse-scored), with a score range of 0 to 9. Participants with scores equal to or greater than the cutoff score (HRS ≥ 3, ELSA ≥ 3, SHARE ≥ 4, CHARLS ≥ 10, and MHAS ≥ 5) were coded as experiencing depressive symptoms, while others were coded as without such condition [32].

The study considered several covariates, including demographic and socioeconomic characteristics, as well as social interaction. Specifically, the study included gender, age, retirement status, education, household wealth, social activities involvement, and weekly contact with children.

### Statistical analysis

We described demographic characteristics as mean and 25th and 75th percentiles for continuous variables, and relative frequencies for categorical variables. To analyze the intercorrelation of repeated measures within each cohort, we used generalized estimating equation (GEE) models with a Poisson distribution and exchangeable correlation structure. We calculated incidence rate ratios (IRRs) and 95% confidence intervals (CIs) with robust sandwich standard errors (SEs). A causal directed acyclic graph (DAG) was used to identify covariates in the minimal sufficient adjustment set (MSAS). We used 3 models to explore the association between digital exclusion and depressive symptoms. Model 1 was unadjusted, and model 2 was adjusted for gender and age. Model 3 was further adjusted for retirement status, education, household wealth, social activities, and contact with children, building on model 2.

Subgroup analyses were conducted to examine the heterogeneity of the association based on weekly contact with children and household wealth quintiles. Furthermore, several sensitivity analyses were performed to assess the robustness of this relationship. First, participants lost to follow-up were excluded. Second, those who had depressive symptoms at baseline were excluded. Third, the analysis was repeated using continuous CES-D or EURO-D scores.

## Results

Our analysis included 122,242 participants (262,391 person-waves) from 5 cohorts spanning the years 2010 to 2018. The median age of the participants was 70 years, with a gender distribution of 45% male and 55% female.

**Fig. 1.**
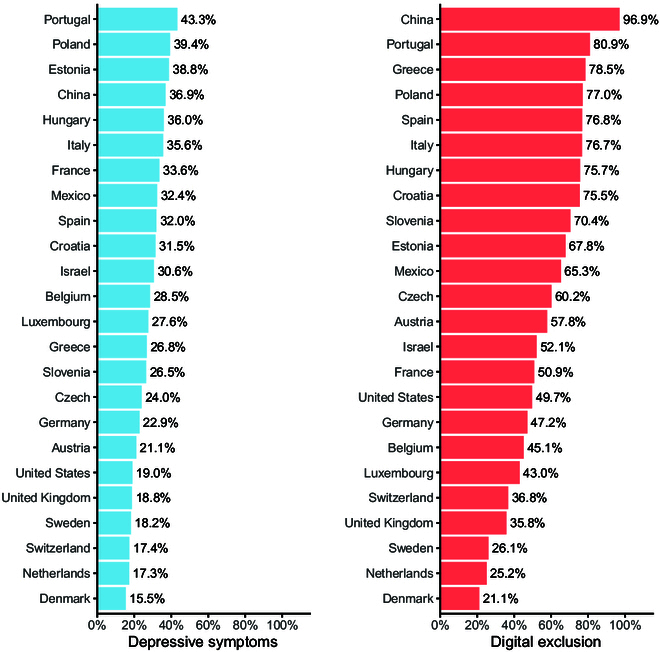
Pooled prevalence of depressive symptoms and digital exclusion by country. Data source: Health and Retirement Study (HRS), English Longitudinal Study of Aging (ELSA), Survey of Health, Ageing and Retirement in Europe (SHARE), China Health and Retirement Longitudinal Study (CHARLS), and Mexican Health and Aging Study (MHAS).

Across the various countries, there was a notable variation in the prevalence of depressive symptoms and digital exclusion. Portugal has the highest proportion of depressive symptoms at 43.3%, while Denmark has the lowest at 15.5%. Digital exclusion was most prevalent in China, where 96.9% of older adults lacked internet access, compared to Denmark, where only 21.1% were digitally excluded (Fig. 1). Examining the cohorts individually, we observed that depressive symptoms were 18.8% in HRS, 18.0% in ELSA, 27.6% in SHARE, 37.6% in CHARLS, and 32.5% in MHAS.

When exploring the association between digital exclusion and depressive symptoms, we found that older adults who were digitally excluded consistently experienced a higher incidence of depressive symptoms compared to their connected peers. This relationship persisted even after adjusting for various demographic and socioeconomic factors, including gender, age, retirement status, education, household wealth, social activities, and contact with children. Although the strength of the association slightly diminished as more covariates were included, it remained statistically significant across all models (Table).

**Table.** Association between digital exclusion and depressive symptoms

	Model 1			Model 2			Model 3		
	IRR	95% CI	*P* value	IRR	95% CI	*P* value	IRR	95% CI	*P* value
HRS	1.76	(1.66–1.87)	<0.001	1.83	(1.72–1.94)	<0.001	1.37	(1.28–1.47)	<0.001
ELSA	1.71	(1.61–1.80)	<0.001	1.58	(1.49–1.67)	<0.001	1.32	(1.23–1.41)	<0.001
SHARE	1.74	(1.70–1.77)	<0.001	1.55	(1.52–1.59)	<0.001	1.30	(1.27–1.33)	<0.001
CHARLS	1.97	(1.74–2.22)	<0.001	1.93	(1.70–2.19)	<0.001	1.62	(1.38–1.91)	<0.001
MHAS	1.44	(1.38–1.51)	<0.001	1.43	(1.37–1.49)	<0.001	1.31	(1.26–1.37)	<0.001

HRS, Health and Retirement Study; ELSA, English Longitudinal Study of Aging; SHARE, Survey of Health, Ageing and Retirement in Europe; CHARLS, China Health and Retirement Longitudinal Study; MHAS, Mexican Health and Aging Study

Model 1 was unadjusted. Model 2 was adjusted for gender and age. Model 3 was further adjusted for retirement status, education, household wealth, social activities, and contact with children, building on model 2.

In the MSAS-adjusted model 3, the impact of digital exclusion on depressive symptoms was consistent across all cohorts: Digitally excluded older adults in HRS had a depressive symptom incidence rate 1.37 times higher (95% CI, 1.28 to 1.47) than those with internet access. Similar trends were observed across other cohorts, with participants in ELSA having an IRR of 1.32 (95% CI, 1.23 to 1.41), 1.30 in SHARE (95% CI, 1.27 to 1.33), 1.62 in CHARLS (95% CI, 1.38 to 1.91), and 1.31 in MHAS (95% CI, 1.26 to 1.37), all with *P* values lower than 0.001.

**Fig. 2.**
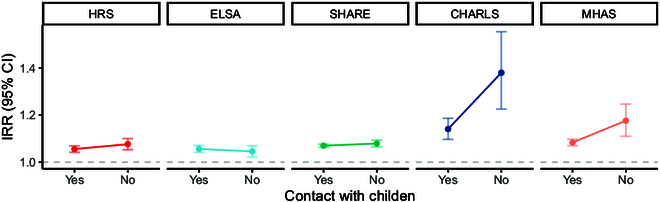
Association between digital exclusion and depressive symptoms by weekly contact with children.

**Fig. 3.**
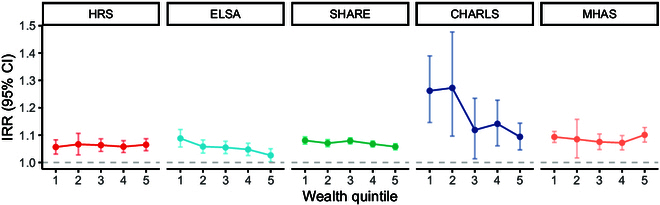
Association between digital exclusion and depressive symptoms across wealth quintiles. Wealth quintile 1 represents the group with the lowest wealth level, and 5 indicates the highest.

Further analysis revealed that the negative effect of digital exclusion on depressive symptoms was more pronounced among older people who do not have regular weekly contact with their children. This pattern was particularly evidence in the HRS, SHARE, CHARLS, and MHAS cohorts (Fig. 2). Additionally, when examining the role of wealth, the association between digital exclusion and depressive symptoms was strongest among those in the lower wealth quintiles across all cohorts. The most significant association was observed in the lowest 2 quintiles in CHARLS (Fig. 3).

To ensure the robustness of our findings, we conducted sensitivity analyses. After excluding participants lost to follow-up or reanalyzing the data using continuous depressive symptom scores instead of a binary outcome, these analyses yielded consistent results, further supporting the significant associations between digital exclusion and depressive symptoms (Table S4).

## Discussion

This study represents the first cross-cultural, longitudinal analysis derived from 5 large and comparative cohort studies to explore the relationship between digital exclusion and depressive symptoms in older people. The size and representativeness of the 5 cohorts support the generalizability of our findings. By including data from 24 countries across 3 continents, this study supports existing research showing that older people who are excluded from the internet are more likely to experience depressive symptoms [7,8].

In general, 2 hypotheses were tested in the present study. First, we found a higher IRR of depressive symptoms among digitally disconnected older adults compared to those digitally included in both HICs (HRS, ELSA, and SHARE) and LMICs (CHARLS and MHAS).

In China, digital exclusion remains relatively high in the older population (97.0% in CHARLS). Older adults are less likely to use computers and the internet than younger groups, despite the potential benefits, such as more access to health information and better management of physical and cognitive decline, loneliness, and social isolation [15]. Previous research has identified barriers such as lack of interest, cost, complexity, and ergonomic limitations that hindered frequent use among older adults [33]. These barriers, leading to difficulties in health management, deserve further attention.

Several possible explanations exist for the association between digital exclusion and depression in older adults. Internet use can promote social participation among older adults, thereby reducing their depressive symptoms [8,12]. In addition, internet use can reduce depressive symptoms in old age by promoting social networks, such as email, chat, and other communication for social contact [8]. Lastly, digital use is associated with a decrease in depressive symptoms through the promotion of self-esteem [34].

Previous studies have identified a positive correlation between depressive symptoms and online social engagement among older adults [35,36]. However, given the diverse ways in which individuals currently utilize information and communication technologies (ICTs), it is imperative to develop measures that represent specific types of usage rather than aggregating them indiscriminately. We advocate for future research to incorporate more detailed inquiries into specific online activities of older adults. This approach will provide a clearer understanding of which online behaviors, such as viewing family photos or seeking information, are most pertinent to their mental health.

Older people who have weekly contact with children would experience a lower increase in the risk of depressive symptoms due to digital exclusion compared to those without weekly contact in all 5 cohorts except in England. Based on the theory of figurative cultures [37], the younger generation may have a responsibility to train the older generation, as the rapid changes in information technology and social media have been referred to as pre-figurative culture [38]. Intergenerational support is commonly regarded as a facet of family or social support [39]. Zhou [40] introduced the concept of “digital back-feeding”, acknowledging the disparities in learning and cognition between older and younger generations. Older adults are generally less inclined than younger individuals to embrace change, including the adoption of new media technologies. In the current era of new media, particularly with the widespread adoption of smartphone technology, the phenomenon of children assisting their parents with internet and smartphone usage is characterized as “digital back-feeding”. It is hypothesized that an increase in the frequency of family feedback is positively correlated with enhanced parent–child relationship harmony and elevated levels of happiness among older adults. Older people who weekly contact with children contribute to the occurrence of digital feeding, which is associated with a lower increase in the risk of depressive symptoms. However, the evidence on this topic is mixed. Some studies suggest that conflicted or distant intergenerational relationships are associated with poorer mental health than strong or close intergenerational relationships [17,41], providing evidence for the socioemotional selectivity theory that emotional needs are increased in older individuals [42], but others did not support it [43].

Addressing digital exclusion requires the coordinated efforts of government, society, and community. Government and relevant departments should ensure that age-friendly smart terminal products, such as mobile phones, incorporate features like large screens, large fonts, high volume, substantial battery capacity, and user-friendly operation. It is imperative to establish standardized and publicly accessible service platforms for testing and certifying intelligent and health-oriented terminal equipment for the older adults. This initiative will enhance the capabilities in the design, research and development, testing, and certification of products tailored for the aging population. Furthermore, advocating for a digitally inclusive society can improve digital literacy among older people. Universities catering to the older adults, along with older adult service organizations and community education institutions, both online and offline, play a crucial role in enhancing the technological proficiency of older adults.

The associations between the digital exclusion and depressive symptoms were most pronounced in the groups with lower wealth quintiles across all 5 cohorts. The most significant association was observed in the lowest 2 quintiles in CHARLS. As a form of social inequality, digital exclusion is essentially a consequence of poverty. Internet access and usage are significantly hindered by income poverty [44,45]. Our findings support the second hypothesis across all 5 cohorts.

Access to the internet should be prioritized over other goods and opportunities for the elderly population. Numerous studies have demonstrated that internet access can enhance the well-being of older adults [46,47]. This has been particularly evident during the COVID-19 pandemic, when such access has become an essential component of their daily lives, encompassing activities such as email and messaging [48]. Furthermore, internet access can support the elderly in maintaining their independence, alleviating depression, and improving overall well-being [49]. Internet usage among older adults enhances interpersonal interactions, fosters social inclusion, and reduces social isolation and loneliness [50]. Moreover, there is a significant correlation between internet usage and civic engagement [51,52]. The elderly are also included in this consideration.

This study makes important contributions. First, we use a cross-cultural and longitudinal study design to demonstrate the generalizability of our findings, as the total sample includes 5 cohorts from 24 countries, both HICs and LMICs, across 3 continents. Second, our findings suggest that limited weekly interaction with children and lower wealth quintile households may exacerbate the negative impact of digital exclusion on depression in older adults. This highlights the importance of improving income level, fostering close intergenerational relationships, and expanding internet access.

Some limitations should be noted. First, self-reported measures are subject to recall bias and may not accurately reflect actual behaviors. Second, the nuances in the measurement of digital exclusion might not fully capture the broader and more consistent use of digital technologies. The different levels of measurement—individual level in HRS, ELSA, SHARE, and CHARLS versus household level in MHAS—could introduce comparison bias across the datasets. Third, the sensitivity analysis suggests that preexisting depressive symptoms may be a strong risk factor for continued or exaggerated depressive symptoms. Future study might address this by using survival analysis. Fourth, it may also be relevant for depression outcomes to examine the type, amount, and timing of internet use [53], although such specifics could not be addressed due to a lack of data.

Enabling older adults to fully enjoy the benefits of digital technology and enhance their quality of life in old age is a critical issue that warrants collective societal attention and resolution in the digital era. From a public perspective, both governmental and community involvement are imperative. The government should develop and implement relevant policies to provide institutional support for older adults’ internet usage. Concurrently, community organizations should intensify efforts to promote internet safety awareness and proactively organize training sessions on internet use. Furthermore, several practical implications are proposed. First, it is essential to establish a digitally inclusive aging society, ensuring that older adults have equitable access to digital information and services. Second, attention must be directed toward addressing the depressive symptoms associated with digital exclusion among older adults. Targeted interventions should be developed and implemented to mitigate core depressive symptoms in this demographic.

## Conclusion

A notable proportion of older adults, particularly in China, lack access to the internet. This phenomenon of digital exclusion is associated with an increased likelihood of experiencing depressive symptoms in older adults. Notably, this association between the digital exclusion and depressive symptoms is particularly pronounced among those who have limited communication with their offspring and lower wealth quintile households. Prioritizing the provision of internet access to older populations may help reduce the risks of depression symptoms, particularly among vulnerable groups with limited family support and with lower wealth level.

## Ethical Approval

We utilized de-identified data from public databases, including HRS, ELSA, SHARE, CHARLS, and MHAS. The ethical approval was covered by the original surveys and was not necessary for the present study. The HRS, ELSA, SHARE, CHARLS, and MHAS participants participated voluntarily and gave informed consent before enrolling.

## Data Availability

The datasets used and/or analysis during the current study are available from the corresponding author on reasonable request.
