# Supplementary material for: Digital Exclusion and Depressive Symptoms among Older People: Findings from Five Aging Cohort Studies across 24 Countries
Source: Health Data Sci. 2025 Jan 10;5:0218. doi: 10.34133/hds.0218 (PMC11717435; doi:10.34133/hds.0218)
Supplement: Supplementary 1 — Tables S1 to S5 [file hds.0218.f1.docx]

Table S1 Included countries in each cohort

| Cohort | Country |
| --- | --- |
| HRS | United States |
| ELSA | United Kingdom |
| SHARE | Austria, Belgium, Croatia, Czech, Denmark, Estonia, France, Germany, Greece, Hungary, Israel, Italy, Luxembourg, Netherlands, Poland, Portugal, Slovenia, Spain, Sweden, Switzerland |
| CHARLS | China |
| MHAS | Mexico |

HRS: Health and Retirement Study; ELSA: English Longitudinal Study of Ageing; SHARE: Survey of Health, Ageing and Retirement in Europe; CHARLS: China Health and Retirement Longitudinal Study; MHAS: Mexican Health and Aging Study.

Table S2 Comparison between included and excluded samples based on missing variables

|  | HRS | | | ELSA | | | SHARE | | | CHARLS | | | MHAS | | |
| --- | --- | --- | --- | --- | --- | --- | --- | --- | --- | --- | --- | --- | --- | --- | --- |
|  | Included | Excluded | P value | Included | Excluded | P value | Included | Excluded | P value | Included | Excluded | P value | Included | Excluded | P value |
| N | 24024 | 1294 |  | 26866 | 4701 |  | 158244 | 2541 |  | 26018 | 7966 |  | 27239 | 645 |  |
| Age, median (Q1, Q3) | 72 (65, 78) | 75 (66, 83) | <0.001 | 69 (64, 76) | 72 (66, 79) | <0.001 | 70 (65, 77) | 71 (65, 79) | <0.001 | 67 (63, 72) | 68 (63, 75) | <0.001 | 69 (65, 76) | 69 (64, 75) | 0.019 |
| Gender |  |  | 0.019 |  |  | <0.001 |  |  | 0.77 |  |  | 0.015 |  |  | <0.001 |
| Male | 41.4% | 44.7% |  | 46.3% | 40.3% |  | 44.5% | 44.8% |  | 50.0% | 48.4% |  | 44.3% | 57.7% |  |
| Female | 58.6% | 55.3% |  | 53.7% | 59.7% |  | 55.5% | 55.2% |  | 50.0% | 51.6% |  | 55.7% | 42.3% |  |
| Education |  |  | <0.001 |  |  | <0.001 |  |  | <0.001 |  |  | <0.001 |  |  | 0.2 |
| Less than upper secondary | 16.1% | 30.3% |  | 25.1% | 43.5% |  | 42.5% | 54.8% |  | 93.0% | 90.3% |  | 88.8% | 86.0% |  |
| Upper secondary and vocational training | 59.9% | 53.7% |  | 48.0% | 38.1% |  | 36.5% | 27.2% |  | 5.6% | 7.0% |  | 2.6% | 3.6% |  |
| Tertiary | 24.1% | 16.1% |  | 19.2% | 9.0% |  | 21.0% | 18.0% |  | 1.4% | 2.8% |  | 8.7% | 10.4% |  |
| Others |  |  |  | 7.7% | 9.3% |  |  |  |  |  |  |  |  |  |  |
| Retirement |  |  | 0.02 |  |  | 0.021 |  |  | <0.001 |  |  | <0.001 |  |  | 0.023 |
| No | 33.7% | 36.8% |  | 24.3% | 22.8% |  | 22.3% | 28.1% |  | 54.5% | 45.9% |  | 81.0% | 77.2% |  |
| Yes | 66.3% | 63.2% |  | 75.7% | 77.2% |  | 77.7% | 71.9% |  | 45.5% | 54.1% |  | 19.0% | 22.8% |  |
| Household wealth |  |  | <0.001 |  |  | <0.001 |  |  | <0.001 |  |  | <0.001 |  |  | 0.45 |
| First quintile | 19.8% | 24.1% |  | 14.3% | 22.3% |  | 20.3% | 26.5% |  | 17.0% | 25.2% |  | 39.3% | 36.1% |  |
| Second quintile | 9.7% | 13.2% |  | 18.9% | 25.3% |  | 18.1% | 20.2% |  | 25.4% | 26.6% |  | 4.1% | 6.4% |  |
| Third quintile | 19.5% | 22.2% |  | 20.8% | 21.3% |  | 20.4% | 20.0% |  | 22.6% | 20.2% |  | 17.6% | 16.7% |  |
| Fourth quintile | 23.7% | 20.6% |  | 22.3% | 17.8% |  | 20.4% | 17.5% |  | 18.8% | 14.8% |  | 18.9% | 20.7% |  |
| Fifth quintile | 27.2% | 19.9% |  | 23.8% | 13.3% |  | 20.8% | 15.7% |  | 16.3% | 13.3% |  | 20.0% | 20.2% |  |
| Social activities involvement |  |  | <0.001 |  |  | <0.001 |  |  | <0.001 |  |  | 0.88 |  |  | 0.073 |
| No | 51.8% | 63.4% |  | 65.3% | 70.7% |  | 59.4% | 66.7% |  | 54.8% | 54.9% |  | 62.3% | 65.8% |  |
| Yes | 48.2% | 36.6% |  | 34.7% | 29.3% |  | 40.6% | 33.3% |  | 45.2% | 45.1% |  | 37.7% | 34.2% |  |
| Weekly contact with children |  |  | 0.001 |  |  | <0.001 |  |  | <0.001 |  |  | 0.37 |  |  | 0.11 |
| No | 26.5% | 32.1% |  | 24.5% | 20.5% |  | 14.7% | 21.2% |  | 11.6% | 12.0% |  | 5.8% | 7.3% |  |
| Yes | 73.5% | 67.9% |  | 75.5% | 79.5% |  | 85.3% | 78.8% |  | 88.4% | 88.0% |  | 94.2% | 92.7% |  |
| Depressive symptoms |  |  | <0.001 |  |  | <0.001 |  |  | <0.001 |  |  | <0.001 |  |  | 0.023 |
| No | 81.2% | 75.4% |  | 82.0% | 76.6% |  | 72.4% | 68.6% |  | 62.4% | 65.9% |  | 67.5% | 71.8% |  |
| Yes | 18.8% | 24.6% |  | 18.0% | 23.4% |  | 27.6% | 31.4% |  | 37.6% | 34.1% |  | 32.5% | 28.2% |  |
| Digital exclusion |  |  | <0.001 |  |  | <0.001 |  |  | <0.001 |  |  | <0.001 |  |  | <0.001 |
| No | 51.3% | 30.0% |  | 66.4% | 49.2% |  | 43.7% | 34.9% |  | 2.8% | 4.0% |  | 34.6% | 47.1% |  |
| Yes | 48.7% | 70.0% |  | 33.6% | 50.8% |  | 56.3% | 65.1% |  | 97.2% | 96.0% |  | 65.4% | 52.9% |  |

HRS: Health and Retirement Study; ELSA: English Longitudinal Study of Ageing; SHARE: Survey of Health, Ageing and Retirement in Europe; CHARLS: China Health and Retirement Longitudinal Study; MHAS: Mexican Health and Aging Study. Q1: 25^th^ percentiles. Q3: 75^th^ percentiles.

Table S3 Proportion of missing data

|  | HRS | ELSA | SHARE | CHARLS | MHAS |
| --- | --- | --- | --- | --- | --- |
| N | 25,318 | 31,567 | 160,785 | 33,984 | 27,884 |
| Digital exclusion | 0.17% | 2.44% | 0.06% | 0.69% | 1.32% |
| Depressive symptoms | 2.09% | 0.38% | 0% | 4.45% | 0.05% |
| Gender | 0% | 0% | 0% | 0.02% | 0% |
| Age | 0% | 0% | 0% | 1.02% | 0.09% |
| Retirement status | 0.10% | 0.01% | 0.20% | 2.29% | 0.27% |
| Education level | 0.02% | 0.41% | 0% | 0.07% | 0.90% |
| Wealth quintiles | 0% | 1.17% | 0% | 20.40% | 0.13% |
| Social activities involvement | 0.58% | 10.45% | 0.36% | 2.55% | 0.07% |
| Weekly contact with children | 2.42% | 2.66% | 1.18% | 0.44% | 0% |

HRS: Health and Retirement Study; ELSA: English Longitudinal Study of Ageing; SHARE: Survey of Health, Ageing and Retirement in Europe; CHARLS: China Health and Retirement Longitudinal Study; MHAS: Mexican Health and Aging Study.

Table S4 Sensitivity analysis

|  | Sensitivity 1 | | | Sensitivity 2 | | | Sensitivity 3 | | |
| --- | --- | --- | --- | --- | --- | --- | --- | --- | --- |
|  | IRR | 95% CI | P value | IRR | 95% CI | P value | IRR | 95% CI | P value |
| HRS | 1.33 | (1.23 - 1.45) | <0.001 | 1.23 | (1.10 - 1.38) | <0.001 | 1.29 | (1.23 - 1.34) | <0.001 |
| ELSA | 1.23 | (1.12 - 1.34) | <0.001 | 0.89 | (0.79 - 1.01) | 0.067 | 1.23 | (1.18 - 1.29) | <0.001 |
| SHARE | 1.23 | (1.15 - 1.32) | <0.001 | 1.01 | (0.96 - 1.05) | 0.825 | 1.17 | (1.16 - 1.18) | <0.001 |
| CHARLS | 1.64 | (1.34 - 2.01) | <0.001 | 1.22 | (0.92 - 1.61) | 0.173 | 1.24 | (1.17 - 1.33) | <0.001 |
| MHAS | 1.29 | (1.23 - 1.36) | <0.001 | 1.03 | (0.94 - 1.13) | 0.535 | 1.14 | (1.12 - 1.17) | <0.001 |

HRS: Health and Retirement Study; ELSA: English Longitudinal Study of Ageing; SHARE: Survey of Health, Ageing and Retirement in Europe; CHARLS: China Health and Retirement Longitudinal Study; MHAS: Mexican Health and Aging Study.

Sensitivity Analysis 1 excluded participants lost to follow-up. Sensitivity Analysis 2 excluded those with baseline depressive symptoms. Sensitivity Analysis 3 used scores of depressive symptoms as continuous variables as outcome.

Table S5 Subgroup analysis of wealth quintiles and contact with children

| Cohort | Variable | IRR | 95% CI | P value |
| --- | --- | --- | --- | --- |
| HRS | Wealth Quintile 1 | 1.056 | (1.03, 1.083) | <0.001 |
| HRS | Wealth Quintile 2 | 1.066 | (1.028, 1.106) | 0.001 |
| HRS | Wealth Quintile 3 | 1.063 | (1.040, 1.087) | <0.001 |
| HRS | Wealth Quintile 4 | 1.058 | (1.036, 1.08) | <0.001 |
| HRS | Wealth Quintile 5 | 1.065 | (1.043, 1.087) | <0.001 |
| ELSA | Wealth Quintile 1 | 1.088 | (1.056, 1.12) | <0.001 |
| ELSA | Wealth Quintile 2 | 1.058 | (1.034, 1.082) | <0.001 |
| ELSA | Wealth Quintile 3 | 1.055 | (1.032, 1.078) | <0.001 |
| ELSA | Wealth Quintile 4 | 1.047 | (1.025, 1.07) | <0.001 |
| ELSA | Wealth Quintile 5 | 1.026 | (1.002, 1.05) | 0.033 |
| SHARE | Wealth Quintile 1 | 1.080 | (1.068, 1.093) | <0.001 |
| SHARE | Wealth Quintile 2 | 1.070 | (1.058, 1.083) | <0.001 |
| SHARE | Wealth Quintile 3 | 1.079 | (1.068, 1.09) | <0.001 |
| SHARE | Wealth Quintile 4 | 1.068 | (1.057, 1.078) | <0.001 |
| SHARE | Wealth Quintile 5 | 1.057 | (1.047, 1.068) | <0.001 |
| CHARLS | Wealth Quintile 1 | 1.262 | (1.146, 1.39) | <0.001 |
| CHARLS | Wealth Quintile 2 | 1.273 | (1.096, 1.477) | 0.002 |
| CHARLS | Wealth Quintile 3 | 1.119 | (1.014, 1.235) | 0.026 |
| CHARLS | Wealth Quintile 4 | 1.141 | (1.061, 1.228) | <0.001 |
| CHARLS | Wealth Quintile 5 | 1.094 | (1.046, 1.144) | <0.001 |
| MHAS | Wealth Quintile 1 | 1.093 | (1.073, 1.114) | <0.001 |
| MHAS | Wealth Quintile 2 | 1.085 | (1.016, 1.158) | 0.015 |
| MHAS | Wealth Quintile 3 | 1.075 | (1.047, 1.104) | <0.001 |
| MHAS | Wealth Quintile 4 | 1.072 | (1.046, 1.098) | <0.001 |
| MHAS | Wealth Quintile 5 | 1.101 | (1.074, 1.128) | <0.001 |
| HRS | No contact with children | 1.077 | (1.053, 1.101) | <0.001 |
| HRS | Contact with children | 1.056 | (1.042, 1.07) | <0.001 |
| ELSA | No contact with children | 1.046 | (1.022, 1.071) | <0.001 |
| ELSA | Contact with children | 1.056 | (1.042, 1.071) | <0.001 |
| SHARE | No contact with children | 1.079 | (1.065, 1.094) | <0.001 |
| SHARE | Contact with children | 1.070 | (1.064, 1.077) | <0.001 |
| CHARLS | No contact with children | 1.380 | (1.225, 1.554) | <0.001 |
| CHARLS | Contact with children | 1.141 | (1.097, 1.187) | <0.001 |
| MHAS | No contact with children | 1.176 | (1.11, 1.247) | <0.001 |
| MHAS | Contact with children | 1.084 | (1.07, 1.098) | <0.001 |

HRS: Health and Retirement Study; ELSA: English Longitudinal Study of Ageing; SHARE: Survey of Health, Ageing and Retirement in Europe; CHARLS: China Health and Retirement Longitudinal Study; MHAS: Mexican Health and Aging Study.
